# VISUALIZING VETERANS’ CARE: UNDERSTANDING THE BREADTH OF OLDER VETERANS’ PAID AND FAMILY SUPPORT NETWORKS

**DOI:** 10.1093/geroni/igad104.1521

**Published:** 2023-12-21

**Authors:** Emily Franzosa, Imran Ali

**Affiliations:** Icahn School of Medicine at Mount Sinai, New York City, New York, United States; The New Jewish Home, New York City, New York, United States

## Abstract

Many older veterans rely on a complex network of paid and unpaid caregivers to age safely at home. Medical records often only capture a primary family caregiver, giving medical teams a limited picture of veterans’ supports at home as well as plans for contingency care. We tested a novel visual tool, care mapping, to better understand the scope of veterans’ formal and informal support. We created collaborative care maps with 10 dyads/triads of veterans age 65+ eligible for VA-paid home care and their primary paid and family caregivers. We compared the maps to veterans’ electronic medical records (EMRs) to explore care network structure, function and adequacy. Care maps identified substantially more care partners than EMRs, including functional, medical, socioemotional and spiritual supports such as family, paid aides, neighbors, Veterans’ groups, faith communities, local businesses (pharmacies, food delivery), and social media. Veterans, family and paid caregivers described working together closely, with aides serving as a “third arm” to fill care gaps. Care was often mutual, as veterans described financial and emotional support they provided to family members, aides and their families, and friends. Participants also described these networks as dynamic and fluid, changing based on care needs and care partners’ availability and skills. Identifying the range of support Veterans receive from family, paid aides, and their community may better help medical teams understand often overlooked community supports and relationships, identify care gaps and tailor home and community-based services where they are most effective and needed.

